# Immunofilaments Are Well Tolerated after Local or Systemic Administration in Mice

**DOI:** 10.1021/acsptsci.4c00180

**Published:** 2024-05-03

**Authors:** Lea Weiss, René Classens, Marjolein Schluck, Emilia Grad, Yusuf Dölen, Lieke van der Woude, Dominique van Midden, Lisa Maassen, Kiek Verrijp, Koen van Riessen, Eric van Dinther, Philipp M. Hagemann, Carl G. Figdor, Roel Hammink

**Affiliations:** § Department of Medical BioSciences, Radboudumc, Geert Grooteplein 26, Nijmegen, GA 6525, The Netherlands; ∥ Institute for Chemical Immunology, Nijmegen, GA 6525, The Netherlands; ⊥ Division of Immunotherapy, Oncode Institute, Radboud University Medical Center, Nijmegen, GA 6525, The Netherlands; # Department of Pathology, Radboudum, Geert Grooteplein 10, Nijmegen, GA 6525, The Netherlands

**Keywords:** biomaterials, toxicology, immunogenicity, polyisocyanopeptides, antigen-specific T cells, immunofilaments

## Abstract

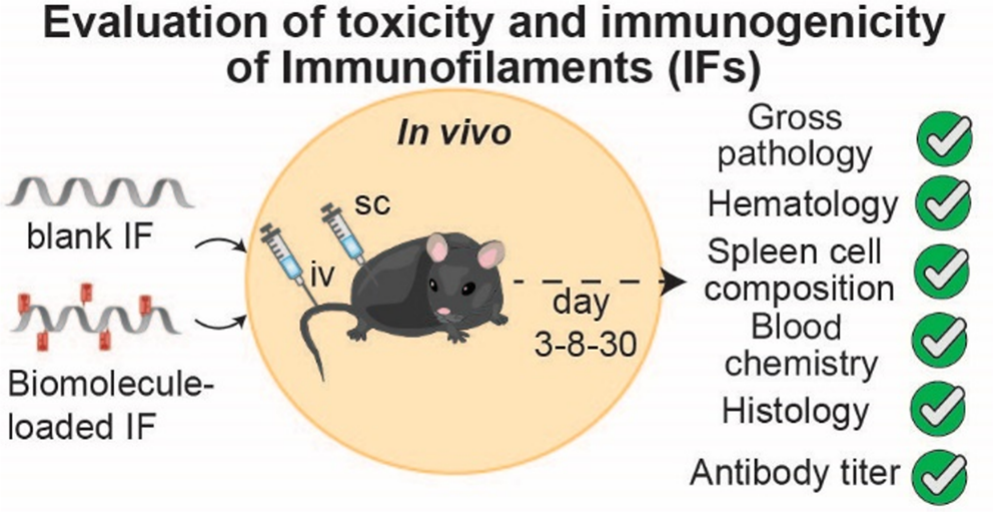

The invention of nanosized biomaterials has paved the way for novel therapeutics that can manipulate cells on a nanoscale. Nanosized immunofilaments (IFs) are synthetic filamentous polymers consisting out of polyisocyanopeptides, which have been recently established as a powerful platform to activate specific immune cells in vivo such that they raise an antitumor immune response. However, toxicological effects or immunogenicity toward the IFs have not yet been investigated. In this study, we evaluated potential toxic or immunogenic effects in C57BL/6 mice upon intravenous or subcutaneous injection of nonfunctionalized IFs or immunostimulatory IFs over 30 days. We here present a detailed analysis of the gross pathology, hematological parameters, blood biochemistry, histology, and antibody-response against the IF backbone. Our results demonstrate that IFs do not induce severe acute or chronic toxicity in mice. After 30 days, we only found elevated IgG-titers in intravenously injected but not subcutaneously injected mice. In summary, we demonstrate that IFs can be administered into a living organism without adverse side effects, thereby establishing the safety of IFs as a therapeutic intervention.

Nanobiomaterials, such as liposomes and polymeric or inorganic nanoparticles, have found widespread application in the medical sector as tools for diagnostics, prophylaxis, and also therapy.^1^ Particularly as cancer therapeutics, nanobiomaterials are ideal delivery devices for small molecules (e.g., chemotherapeutic agents) or biomacromolecules, such as monoclonal antibodies or peptides. Careful manipulation of the chemical or physical properties of the nanobiomaterial yields nanomedicines with improved drug loading capacity, favorable biodistribution to, e.g., the tumor site and specific cell-targeting properties.^2^ At the same time, the off-target toxicities of the payload can be reduced. However, next to the validation of the therapeutic efficacy, detailed investigation of the nanobiomaterial’s pharmacokinetics, toxicity, and immunotoxicity/immunogenicity is a critical step toward clinical approval.^3,4^ Owing to their small size, nanobiomaterials accumulate in the liver, spleen, and kidneys, where they form potentially toxic aggregates and lead to tissue damage.^1^ In addition to the size, geometry, and surface charge as well as chemical modification, deformability and biodegradability can determine the severity of toxic effects or immunogenicity, which can impede further clinical testing.

One innovative nanobiomaterial currently under investigation for its clinical potential is polyisocyanopeptide polymer (PIC). PICs form a stiff helical backbone when isocyanide monomers are polymerized with a nickel perchlorate (Ni(ClO_4_)_2_) catalyst.^5−7^ Hydrogen bonds between the alanines in the side chain further stabilize the helical structure, providing the polymer with its unique semiflexibility that allegedly improves its interaction with cells (Figure 1a).^8−10^ To design polymers for medical use, oligo(ethylene glycol) tails were previously introduced into the side chain, rendering water-soluble polymers. These polymers demonstrated different thermosensitivities depending on the number of ethylene glycols added, providing many possibilities for medical applications.

**Figure 1:**
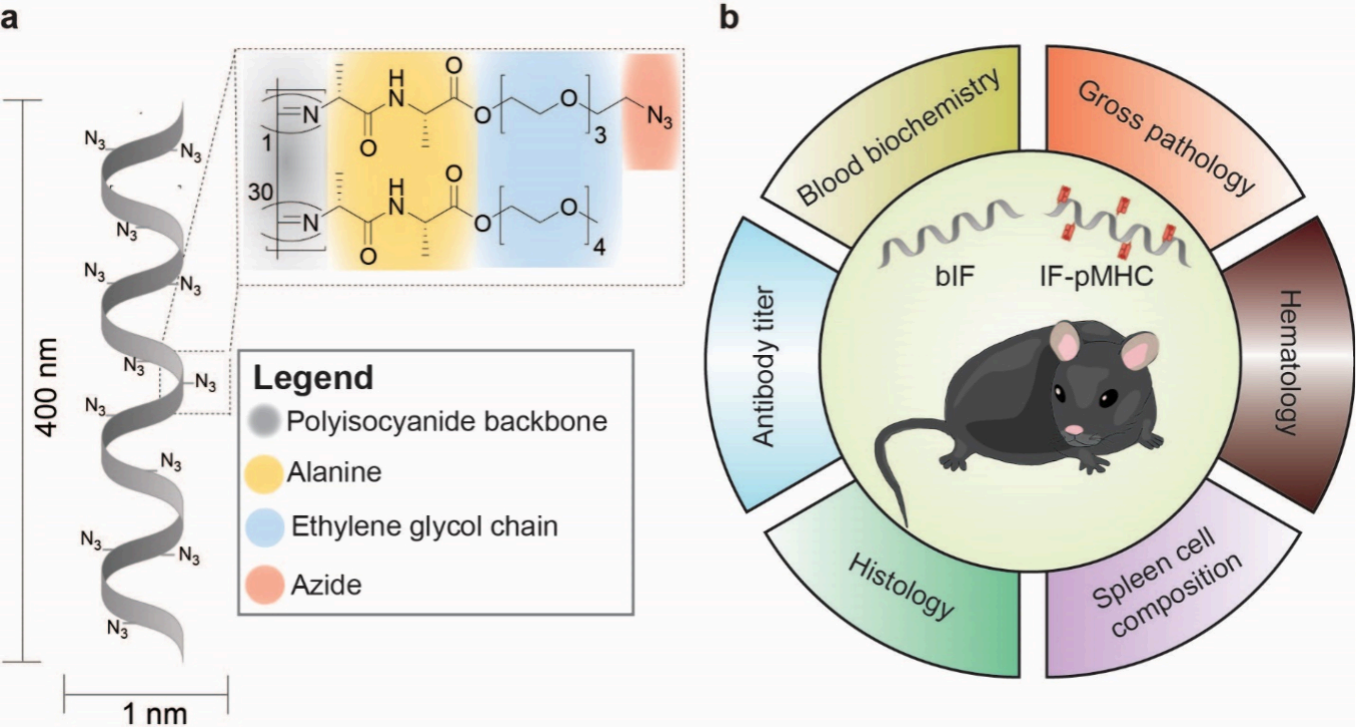
Structure of IFs and overview of the evaluated study parameters. (a) IFs are helical polymers consisting of a polyisocyanide backbone of ∼400 nm length and ∼1 nm width. Two alanines in the side chain form hydrogen bonds parallel to the backbone to stabilize the helix. Tetra-ethylene-glycol chains ensure water solubility. For functionalization with biomolecules, using biorthogonal click chemistry, reactive azide monomers are built in with a 1:30 ratio with methoxy monomers. (b) Overview of the different parameters evaluated after either iv or sc injection of bIF or IF-pMHC in mice.

Triethylene glycol PICs (PIC3eg), for instance, are an ideal means for wound dressings in mice and rabbits given the fact that the hydrogel’s mechanical properties (e.g., strain stiffening) closely mimic a biopolymer network such as collagen.^11−15^ As PIC3eg undergoes transition into a hydrogel above 18 °C, it can be injected in a minimally invasive manner. Additionally, in an immunotherapeutic setting, 3D PIC3eg scaffolds have been successfully applied to deliver preactivated T cells *in vivo*.^16^ Of note, none of the mentioned studies reported any harmful effects of PIC3eg hydrogels in mice or rabbits.

Tetra-ethylene glycol PICs (PIC4eg) are the building blocks of IFs – a next-generation cancer immunotherapeutic platform aimed at harnessing the power of the immune system to fight cancer. The tetra-ethylene glycols sustain the IF in its soluble filamentous form below 42 °C,^15^ allowing it to circulate in the blood for multiple days and to drain to lymphoid organs where it can interact with immune cells.^17^ The azide group in the IF’s side chains enables straightforward functionalization using strain-promoted alkyne azide click reactions (SPAAC), and bicyclononyne (BCN)- or dibenzocyclooctyne (DBCO)-modified biomolecules, such as antibodies or cytokines.^18,19^ Given this modular and versatile design, several different IFs have been created for immune cell stimulation.^16,18−21^ We recently demonstrated that IFs functionalized with peptide-loaded major histocompatibility complex (pMHC) are a powerful tool to trigger tumor-antigen-specific T cells *in vivo*, resulting in reduced tumor growth and prevention of metastases formation.^17^ Using this approach, a laborious, time-intense, and costly *ex vivo* culture of cytotoxic T cells may be omitted, making T cell-based cancer immunotherapies amenable to more patients suffering from cancer.

To put IFs forward as a novel cancer nanomedicine, we sought to gain deeper insight into the toxicological or immunogenic effects of IFs (Figure 1b). In the present study, we assess acute or chronic effects of nonfunctionalized blank IFs (bIFs) or pMHC-functionalized IFs (IF-pMHC) after intravenous (iv) or subcutaneous (sc) injection. Our results demonstrate that the tested doses of IFs within the tested time frame are nontoxic and do not lead to any acute or chronic pathologically relevant alterations. In addition, we evaluate whether the IFs, and particularly the conjugation of an immunostimulatory molecule such as pMHC, promote antibody responses in the presence of adoptively transferred antigen-specific T cells. Although we did not observe an antibody response in the sc group, we report an increase in IgG-titers in the treated iv groups on day 30, indicating some immunogenicity inherent to the current IF format. This response was likely not associated with the stimulation of antigen-specific T cell stimulation as antibodies were found after iv administration for both bIF and IF-pMHC. Collectively, our results highlight the potential of IFs as a therapeutic intervention by validating their safety to be administered *in vivo*.

## Results

### Weight and Temperature

**Figure 2:**
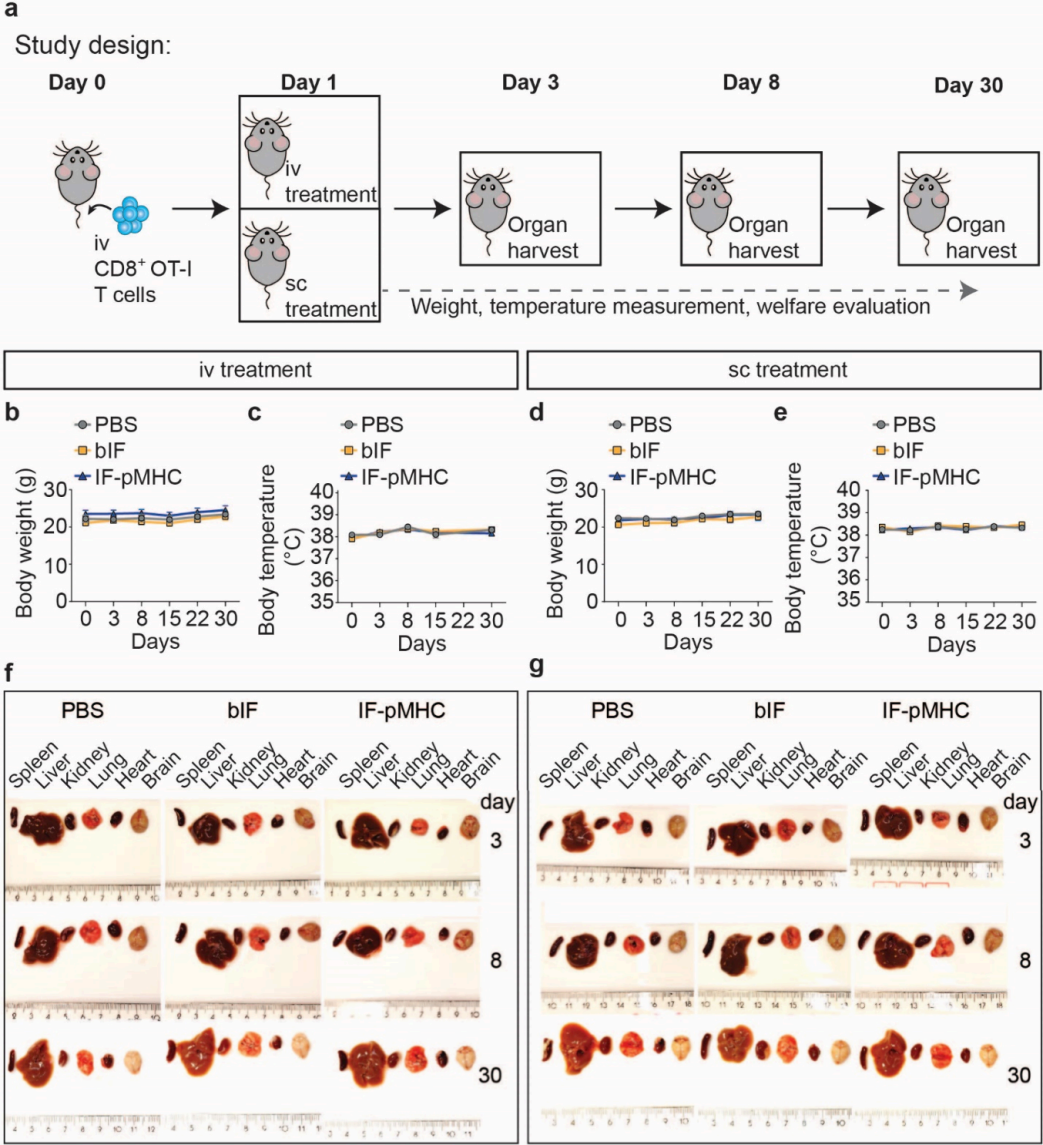
Evaluation of gross pathology during the study period. (a) Overview of study design. C56BL/6 mice were injected intravenously with one million OT-I CD8^+^ T cells on day 0. On day 1, mice received either PBS, bIF, or IF-pMHC iv or sc. *n* = 12 mice per treatment and injection route. Mice were monitored for decrease in welfare, weight, or changes in temperature. On days 3, 8, or 30, 4 mice per treatment group per injection route were sacrificed and organs were harvested for downstream analysis. (b,c) Body weight (g) and body temperature (°C) measured overtime of iv injection group. (d,e) Body weight (g) and body temperature (°C) measured overtime of sc injection group. (f,g) Representative images of organs taken from the iv (f) or sc (g) group. Organs from left to right: spleen, liver, kidney, lung, heart, and brain. *n* = 4.

Previous experiments in mice bearing tumors did not show explicit toxicity by the IF. Yet, we did not systematically study whether IF as a nanobiomaterial is toxic in healthy mice. Our first aim was to assess any acute or long-term severe effects in mice upon iv or sc administration of synthetic IFs. Similar to our earlier *in vivo* study, we iv injected the maximum injectable dose of IF-pMHC and a matching dose of bIF that is possible with the current system (6 μg iv in 200 μL). A lower dose was chosen for sc injection, as we previously showed that a lower dose of IFs sc was effective in reducing sc tumors (1 μg of sc in 100 μL). Sterile PBS was administered as a negative control. To fully understand adverse side effects induced by the IF, we adoptively transferred OT-I CD8^+^ T cells 1 day prior to the administration of treatments (Figure 2a). OT-I CD8^+^ T cells are activated by the IF-pMHC and might foster an immune reaction in mice, which could contribute to the toxicity of the IF.^17^

The first indication of an acute and severe systemic response to a material is sudden weight loss and a rise in body temperature. Over the course of the experiment, we did not observe weight loss in any of the mice, demonstrating that IFs do not affect the general body metabolism (Figure 2b,d). Moreover, we observed a similar body temperature across the different groups, indicating that no acute systemic inflammatory response is raised in mice (Figure 2c,e). We did not observe any abnormal behavior or changes in locomotor activity in mice of the different treatment groups, and overall mice appeared healthy during all sightings. In addition, the sc group receiving bIF or IF-pMHC did not show any redness or abnormal palpable hardening of the tissue at the injection site. On days 3, 8 and 30, we harvested and weighed the organs of all mice and did not find any deviations in organ weights between the groups (Table S1). In addition, optical images of the liver, lung, heart, kidney, spleen, and brain did not show any obvious changes such as hypertrophy, inflammation, or a change in color (Figure 2 f,g). In summary, we report that bIF or IF-pMHCs do not induce any gross pathological alterations in mice until day 30.

### Blood Hematology and Spleen Composition

**Figure 3:**
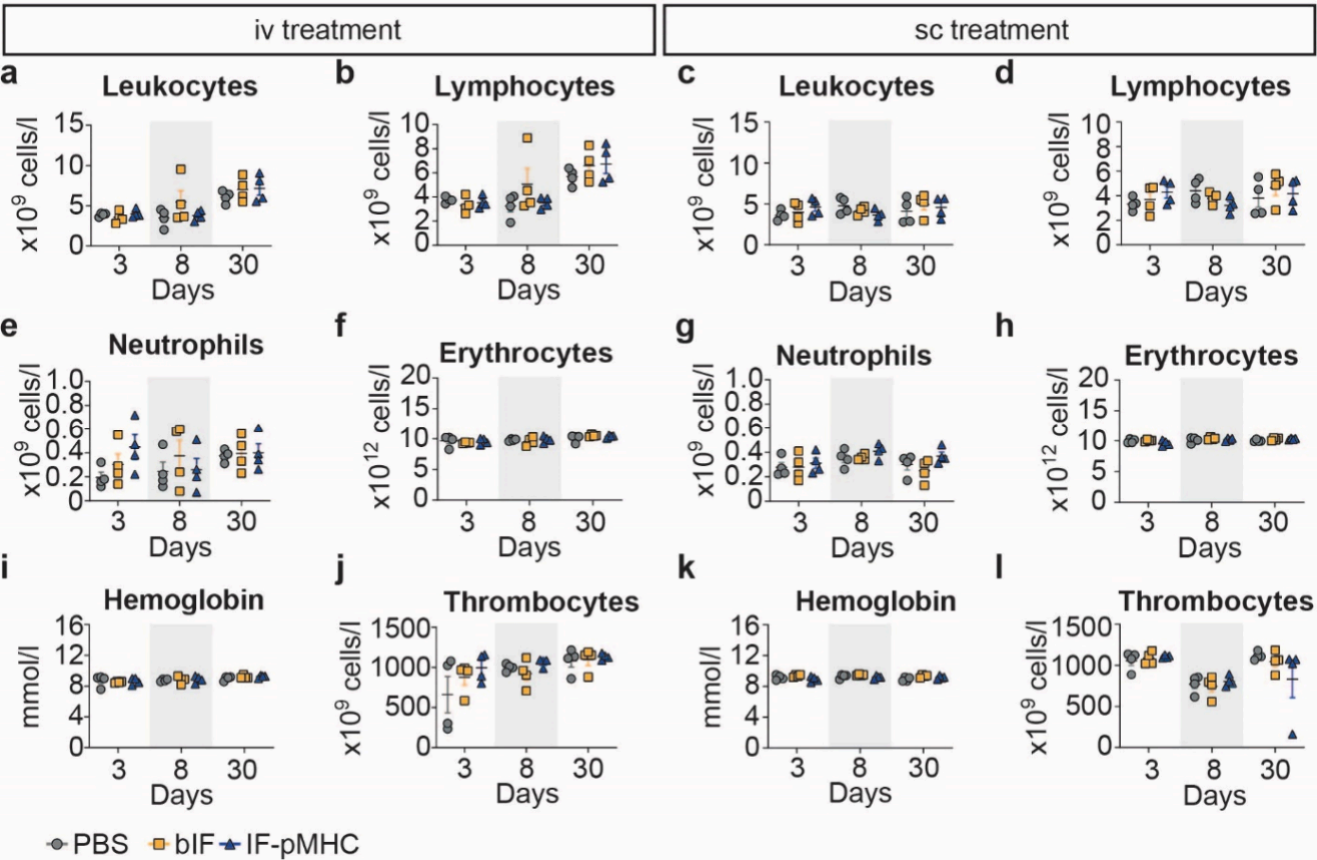
Blood hematological analysis shows no differences between the treatment groups. Hematological parameters were evaluated following iv or sc injection of treatments on days 3, 8, or 30. Statistical analysis was done per day using a nonparametric Kruskal–Wallis test with posthoc Dunn’s multiple comparison test. *n* = 4 mice per group.

On days 3, 8, or 30, part of the mice were euthanized, and blood was collected through retro-orbital bleeding for subsequent evaluation of several hematological parameters. These included the count of leukocytes, lymphocytes, neutrophils, erythrocytes, and thrombocytes as well as the hemoglobin concentration (Figure 3a–l, Figure S1). Most of the measured hematological parameters were in the expected range and did not indicate clinically relevant deviations between the IF-treated groups and the PBS control^22^ (Figure 3a–l, Figure S1). The increase in leukocytes and lymphocytes on day 30 in all treatment groups of the iv group indicates that an external factor might be the cause of that effect (Figure 3a,b). We observed a small transient and nonsignificant increase of neutrophils in the iv IF-pMHC-treated mice on day 3, which was already resolved on day 8 and remained low (day 30) (Figure 3e). Elevated levels of neutrophils are observed during the initial phase of an inflammatory response or during defense from foreign materials;^23^ however, the levels measured on day 3 are well within the range measured in healthy female C57BL/6 mice.^24^ In addition, we noted that the overall count of monocytes is around 10× lower than would be expected in murine blood, indicating that some monocytes were not classified as such or indicating differences in analyzer properties^22,25−27^ (Figure S1q). Next to analyzing the peripheral blood, we also assessed the immune cell composition in the spleen through flow cytometry. Similar to the results from the hematological examination, we did not observe any significant deviations in cell composition from the baseline PBS control (Figures S2 and S3). All identified cell populations remained stable over time and were not influenced by IF treatment.

All in all, the measured hematological parameters and the spleen cell compositions were similar between IF-treated and control mice, demonstrating that IFs are biocompatible.

### Blood Chemistry

**Figure 4:**
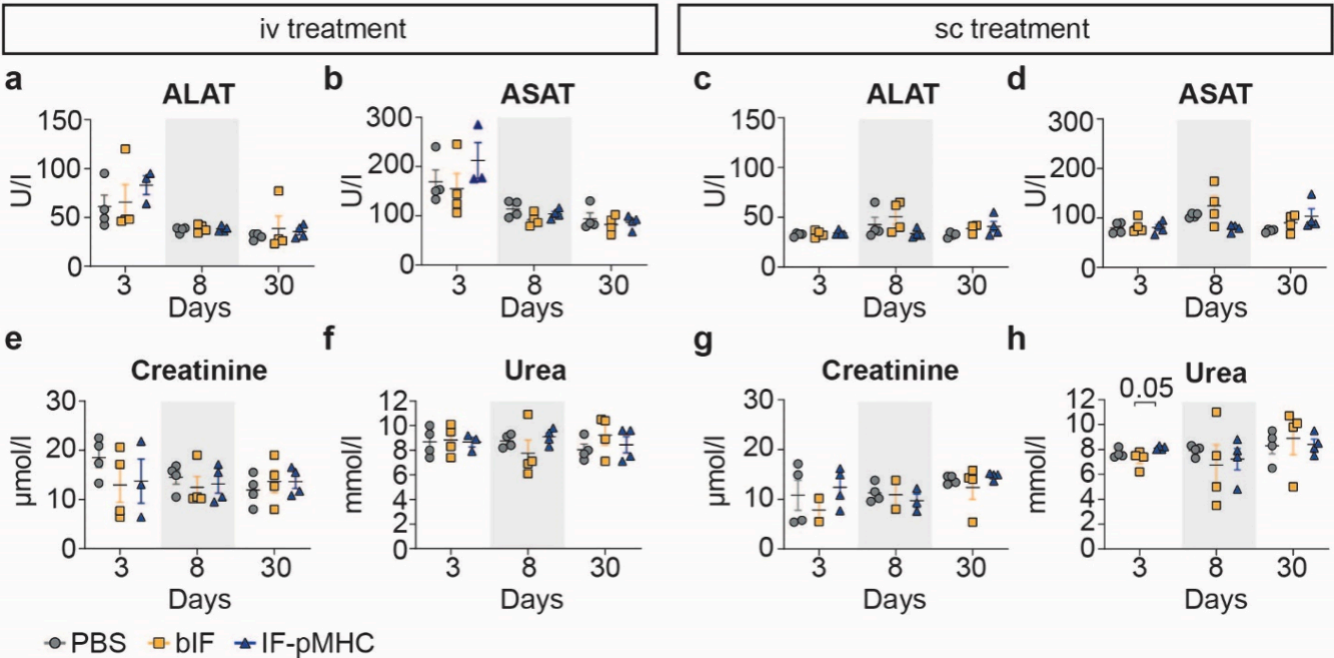
Plasma biochemical analysis shows no nephrotoxicity or hepatotoxicity. (a–d) Liver damage was measured in the blood of mice on days 3, 8, or 30 following iv (a,b) or sc (c,d) treatment with PBS, bIF, or IF-pMHC. (e–h) Kidney damage was measured in the blood of mice on days 3, 8, or 30 following iv (e,f) or sc (g,h) treatment with PBS, bIF, or PIC-pMHC. ALAT: alanine aminotransferase, ASAT: aspartate aminotransferase. Statistical analysis was tested per day using a nonparametric Kruskal–Wallis test with posthoc Dunn’s multiple comparison test. Data are shown as mean ± SEM *n* = 4 mice per group.

Next to accumulation in the spleen, we observed that a majority of IFs accumulate in the liver upon iv injection. Hence, we evaluated whether IFs induce hepatotoxicity and nephrotoxicity in the presence of reactive T cells. By testing the plasma biochemical composition, one can assess the metabolic functionality of the liver (alanine aminotransferase (ALAT), aspartate aminotransferase (ASAT)) and kidneys (urea, creatinine). Elevated levels of ALAT and ASAT are indicators of liver damage,^28^ while altered levels of urea and creatinine can indicate kidney failure. None of the values measured in bIF- or IF-pMHC-treated mice were significantly elevated on any of the measured days compared to the PBS control. We found slightly elevated levels of ALAT and ASAT in all mice euthanized on day 3 compared to day 8 and day 30, which we attribute to some hemolysis that occurred during blood processing (Figure 4a,b). The sc group did not show elevated ALAT or ASAT levels on any of the days (Figure 4c,d). The urea and creatinine measurements did not differ between the treatment groups (Figure 4e–h). Moreover, we measured the C-reactive protein (CRP), which is released during acute inflammatory responses.^29^ We did not find detectable levels of CRP in any of the mice. Altogether, our results highlight that systemic as well as local injection of IFs in the presence of sensitive T cells does not lead to liver or kidney damage.

### Histological Analysis

**Figure 5:**
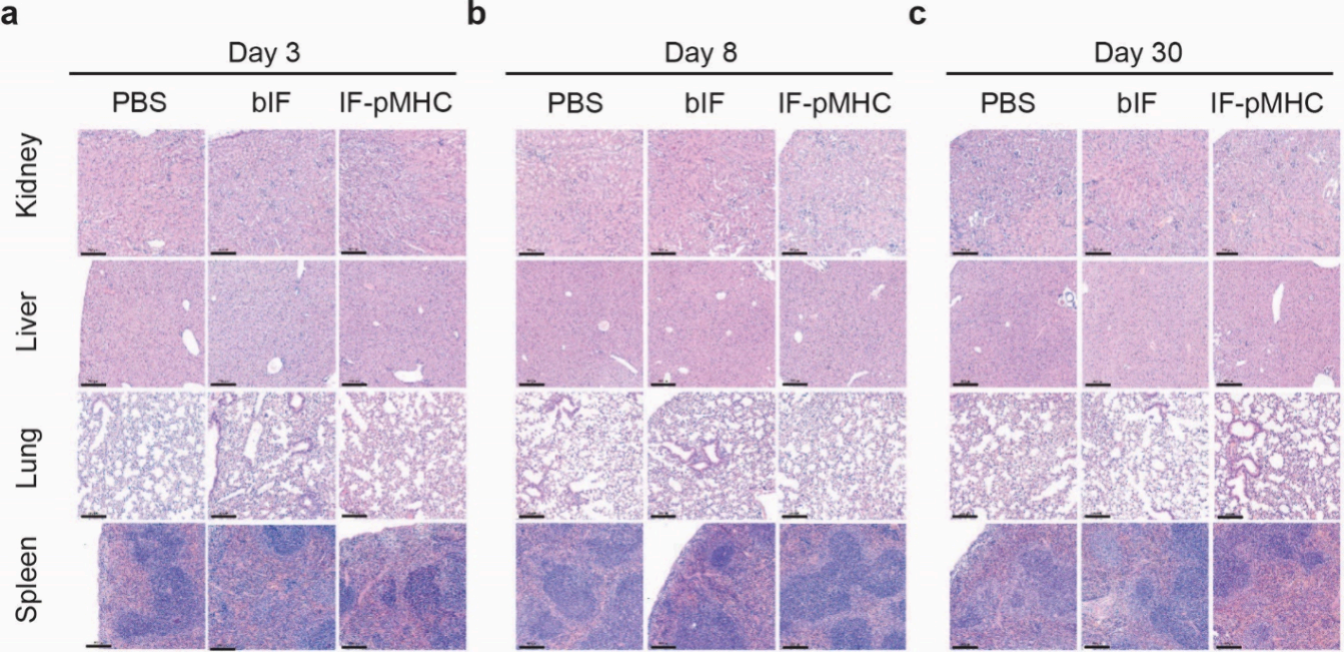
Histological analysis of organs from mice treated iv does not show alterations on tissue level. (a–c) Histological slices of the kidney, liver, lung, and spleen on day 3 (a), day 8 (b), or day 30 (c) from the iv treatment group treated with PBS, bIF, or IF-pMHC. Scale bar is 200 μm.

To further explore any possible toxic effect on tissue level in the iv group, we prepared H&E tissue sections of the kidney, liver, lung, and spleen. All organs were scored independently by two pathologists under blinded conditions. No moderate or severe lesions or changes in the vasculature or hyperplasia were reported (Figure 5a–c). In some mice, minimal immune cell infiltration was observed, which was not specific to IFs (Table S2).

### Antibody Titer against bIF

**Figure 6:**
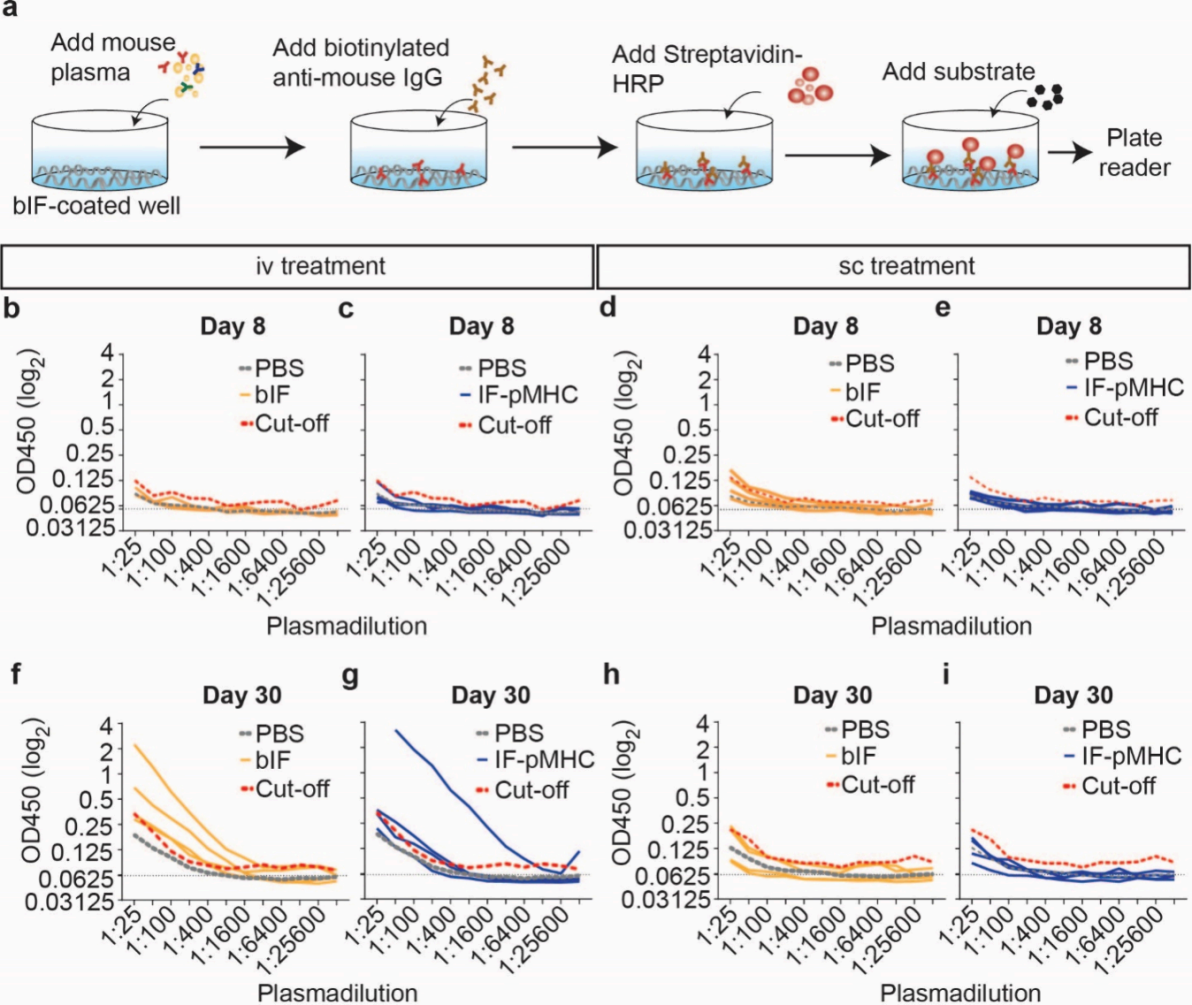
IgG-titers against bIF are elevated in the iv group on day 30 but not the sc group. (a) Setup of sandwich-ELISA to measure IgG-titers against the bIF. (b,c) IgG-titers on day 8 of iv group treated with either bIF (b) or IF-pMHC (c). (d,e) IgG-titers on day 8 of sc group treated with either bIF (d) or IF-pMHC (e). (f,g) IgG-titers on day 30 of the iv group treated with either bIF (f) or IF-pMHC (g). (h,i) IgG-titers on day 30 of the sc group treated with either bIF (h) or IF-pMHC (i). *n* = 4. Each line represents an individual mouse. The gray-dotted line is the average of the PBS control group. The red-dotted line is the cutoff calculated based on the mean of the PBS group times 3× the standard deviation.

To investigate the possibility of an antibody response directed against the bIFs, we performed a sandwich-ELISA using bIF-coated plates onto which we incubated the obtained mouse plasma from the different treatment groups (Figure 6a). Subsequently, we incubated the samples with an antimurine-IgG antibody. None of the mice had elevated IgG-titers on day 8 (Figure 6b–e). After 30 days, we noticed elevated IgG-titers in mice that received IFs iv. 2 out of 4 mice of the bIF-treatment group and 1 out of 4 mice of the IF-pMHC-treatment group had elevated IgG-titers (Figure 6f,g). In contrast, sc injection of bIF or IF-pMHC did not show an elevation in IgG-titers compared with the PBS group on any of the evaluated days (Figure 6h,i).

## Discussion

Careful evaluation of the toxicity and immunogenicity of nanobiomaterials in mice is pivotal before any further evaluation in larger animals or clinical trials should be considered. In this study, we opted to investigate two injection routes of IFs, which was based on our previously published *in vivo* work showcasing the therapeutic efficacy of IFs functionalized with pMHC in tumor mouse models.^17^ Since the aim of the therapy with IF-pMHC is to stimulate antigen-specific T cells *in vivo*, we further simulated a situation in which antigen-specific T cells are present by adoptively transferring OT-I CD8^+^ T cells. Thus, our results reflect not only the toxicological or immunogenic effect of IFs but also the possible immune response of antigen-specific T cells and, consequently, other interacting immune cells.

Based on our previous work,^17^ we observed IF-signal in the liver, lung, and spleen, which are the main accumulation sites of nanomaterials.^1^ Accumulation of IFs at the spleen, liver and lung can be particularly favorable to warrant activation of circulating cytotoxic T cells or to foster antimetastatic responses in the lung or liver, which are predominant sites of metastases.^30,31^ Importantly, our results rule out that accumulation of IFs in the applied doses in these organs is toxic based on histological and blood biochemistry analysis of hepatotoxicity markers. Whereas others found alterations in markers associated with kidney damage upon injection of various nanomaterials, we did not find deviations in urea or creatinine upon injection of IFs.^32−34^ Overall, our results demonstrate that IFs are well tolerated without any toxic side effects, showing that IFs form a suitable nanomedicine platform for cancer therapy in patients.

Many of the commonly used nanomaterials are spherical nanoparticles. In contrast, our IFs are nanometer-scale filamentous polymers with an aspect ratio of 1:400, which likely endows them with different pharmacokinetic and clearance properties from the body compared to spheres.^35^ Previous work suggests that rod-like particles circulate longer in the blood as they cannot be as easily taken up by phagocytes as compared to spherical particles, resulting in higher accumulation in tumor tissues underlying the enhanced retention and permeability effect.^36−39^ In addition, the tetra-ethylene glycol in the side chains of the IFs can further increase the circulation time and reduce protein absorption, as was previously demonstrated for gold nanoparticles.^40,41^ Remarkably, we could detect radioactivity of Indium-labeled IFs for up to 3 days in the blood, suggesting that IFs are not rapidly eliminated through renal clearance.^17^ Given an average polymer length of 400 nm and a width of 1 nm, plus conjugated molecules, that can further increase the hydrodynamic diameter, along with the high accumulation in the liver, supports the hypothesis that the majority of our IFs are eliminated from the body through the hepatobiliary route.^42^ In fact, only particles smaller than 6 nm are eliminated through renal clearance.^43,44^ Next to the IF-signal in the liver, our previous study also showed that CD11b^+^ and CD11c^+^ cells in the spleen (primarily macrophages and dendritic cells) take up the IFs. Henceforth, an alternative route of IF elimination could be clearance from the blood through the mononuclear phagocytic system (MPS). The MPS comprises but is not limited to Kupffer cells in the liver, splenic red pulp and marginal zone macrophages in the spleen, and blood-circulating monocytes.^45^ After uptake by these cells, materials are either enzymatically degraded or remain in the cells for sometimes even longer than six months if degradation is not possible.^43^ Thus, future studies are warranted to investigate in more detail the pharmacokinetics, elimination route, degradation, and intracellular and subcellular localization of IFs upon single and multiple injections. Understanding these parameters will help to guide future development of IFs, ensuring prolonged circulation while preventing long-term accumulation in tissues by tailoring biodegradability.

Furthermore, we evaluated potential immunogenicity against the IF backbone by measuring IgG-titers.^4^ Increased antibody titers are suggested to be responsible for accelerated blood clearance, which can compromise the therapeutic efficacy and can result in adverse effects.^46^ For example, antipolyethylene glycol IgM antibodies have been observed to develop against pegylated nanomaterials or to be already present in healthy individuals, resulting in the introduction of guidelines by the FDA to assess anti-PEG antibodies in individuals receiving pegylated agents.^47,48^ Our results indicate that sc injection is favorable over iv injection due to the lack of any IgG antibodies against the material after 30 days. Previously, it has been shown that increasing the size of liposomes (more than 300 nm) decreases detectable blood levels after sc injection and that only a small amount of liposomes was detectable in the liver.^49^ Others showed that sc injection, independent of the size of the liposome, resulted in lower bioavailability.^50^ Thus, sc injection of IFs might prevent immunogenicity by a slower release into the circulation and lower accumulation into organs where an immune response could take place. An alternative explanation for the occurrence of the antibody response in the iv group but not the sc group could lie in the difference between the injected dose of IF iv or sc. Future studies will elucidate whether the measured antibody titers in the iv group are clinically relevant and trigger hypersensitive reactions or whether IFs are well tolerated, particularly, upon multiple iv injections. A deeper analysis of a possible IF-directed antibody response can pave the way toward an optimized IF format for clinical applications. Given the versatile and modular nature of our IF platform, immune responses could be tailored by additional modification of the polymer backbone or functionalization of the side chains depending on the desired outcome. As such, the therapeutic index, meaning the balance between efficacy and safety of IF can be strengthened further.

## Conclusions

In conclusion, we here present evidence that nonfunctionalized as well as therapeutically effective IFs are nontoxic and biocompatible. Gross pathological parameters as well as hematological, serum biochemical, and histopathological analysis exclude that a one-time injection of IFs either iv or sc at the evaluated doses induces severe toxicity. Ultimately, this study is a pivotal step in the clinical evaluation of IFs as cancer immunotherapeutics.

## Materials and Methods

### Immunofilament Synthesis and Characterization

IFs were prepared as described before.^15,21^ Briefly, isocyanopeptide monomers with nonfunctional methoxy and functional azide groups were polymerized in a 30:1 ratio using a nickel catalyst (1/10,000 ratio), yielding polymers with statistically one azide group every 3.5 nm.^20,51,52^ Next, 60% of the azides were reacted with DBCO-PEG4-biotin according to literature procedures.^53^ The average length of the azide/biotin polymers was determined using atomic force microscopy (AFM, nanoscope III, digital instruments), operated in tapping mode in air. The polymers were dissolved in Milli-Q (10 μg/mL) and drop-casted on freshly cleaved mica for 5 min, after which the sample was dried under a nitrogen flow. From the resulting images, the polymer length was determined using ImageJ.^54^ The average length determined was 407 ± 207 nm, calculated from 161 values. DLS could not be performed on IFs as they are linear thread-like polymers with a semiflexible character, and no adequate models for fitting of the data are available.

### MHC Production

MHCs were prepared as described.^55^ The constructs for the human beta-2-microglobulin (hβ2m) were generously provided by M. Toebes and T.N. Schumacher from the NKI. They were produced as inclusion bodies in *E. coli* BL21(DE3)pLysS using a T7 RNA polymerase/promoter system.^43^ Isolated inclusion bodies were solubilized in a denaturing buffer (8 M urea/100 mM Tris•Cl, pH 8). Hβ2m was prefolded in dialysis against 10 mM Tris•Cl (pH 7) in PBS. To prepare the final MHC complex, hβ2m and heavy chain were dissolved to final concentrations of 6 and 3 mM respectively in folding buffer (100 mM Tris•Cl, pH 8; 400 mM l-arginine; 2 mM EDTA; 5% glycerol; 5 mM reduced glutathione; 0.5 mM oxidized glutathione; protease Inhibitor Cocktail, Roche Diagnostics) with 60 mM template (SIINFEKL, GenScript). The folding reaction mixture was incubated at 10 °C for 5 days. After filtration, concentration, and buffer change to PBS, the complexes were purified via size exclusion chromatography using HiLoad 16/600 Superdex 75pg column (Cytiva). Ready MHC was analyzed using SDS-PAGE and NanoDrop, concentrated, snap-frozen, and stored at −80 °C until further use.

### Functionalization of pMHC

Proteins were functionalized using previously reported protocols.^18,19^ Briefly, pMHC was obtained from the refolding protocol, typically at 0.5–2 mg/mL in PBS (pH 7.4). To functionalize the pMHC, DBCO-PEG4-NHS (click chemistry tools, 100 mM in DMSO) and Atto488-NHS (10 mM in DMSO, AttoTec) were added to the protein stock solutions; typically 3–4 equiv of DBCO and 2.5–3 equiv of dye were used. PMHC was left to react for 4–6 h at 4 °C. After the reaction, pMHC was purified using spin filtration against PBS with spin filters of appropriate size (Amicon). The protein-conjugates were analyzed with NanoDrop (Thermo Fisher Scientific), using the following extinction coefficients for MHCs (95000 M^–1^ cm^–1^), DBCO (12000 M^–1^ cm^–1^), and Atto488 (90000 M^–1^ cm^–1^). Data was measured at 280 nm (protein), 309 nm (DBCO), and the wavelength of the dye. The raw data was corrected for spectral overlap between the different components using correction factors as described.^18^ Typically obtained degrees of labeling (DOL) for DBCO were 0.5–3 and 0.5–2 for dyes.

### Conjugation of pMHC to Immunofilaments

The DBCO/dye-functionalized pMHC was coupled to the IFs using a protocol as described before.^17−19^ Briefly, 100–200 μL of a stock solution of 1 mg/mL biotin/azide polymer was reacted with the required amount of protein in PBS (pH 7.4), typically 0.2–1 equiv of protein irt free azides was used. All reactions were carried out in nonstick Eppendorf tubes (Fisher Scientific), with a final concentration of 0.2–0.25 mg/mL polymer. Reactions were first mixed for 4–5 h at room temperature (rt) followed by incubation at 4 °C overnight. Purification was performed following a literature protocol, using monoavidin resin (Thermo Fisher Scientific).^53^ Per 100 μg of polymer, 1 mL of monoavidin resin was used. After 2× washing of the resin with PBS (pH 7.4), the resin was added to the reaction mixtures and incubated for 1.5–2 h at 4 °C. Next, the resin was washed with 1× PBS-tween (0.1%) and 4–5× PBS. After washing, a solution of 2 mM biotin in PBS was added (300–400 μL) to elute the polymer bioconjugates from the monoavidin resin (1–2 h incubation at 4 °C). Concentrations of the conjugated proteins were determined using fluorescence (Tecan spark 10 M plate reader) with the soluble labeled proteins as the standard. Polymer concentration was determined by using circular dichroism spectroscopy (JASCO J-810), from which a standard curve was inferred to determine the loading amount of coupled proteins. With these concentrations, the average spacing/density of the protein on the polymer could be calculated, using the fact that every monomer is 0.115 nm in size.^56^

### CD8^+^ T Cell Isolation

OT-I CD8^+^ T cells were isolated from OT-I (aka C57BL/6-Tg(TcraTcrb)1100Mjb mice) (Charles River). Briefly, spleens and lymph nodes were harvested and digested in 20 μg/mL Dnase I (Roche, 11284932001) and 1 mg/mL collagenase III (Worthington, LS004182) for 30 min at 37 °C. Organs were put through a 100 μm cell strainer. Erythrocytes were eliminated by in-house ammonium-chloride-potassium (ACK) lysis buffer. CD8^**+**^ T cells were isolated by negative selection using a CD8^+^ T cell isolation kit (Miltenyi, 130–104–075). Cells were diluted to the desired cell concentration in sterile saline.

### Mice

All mice used in the experiments were housed at the Central Animal Laboratory (Nijmegen, The Netherlands) in accordance with European legislation. All conducted protocols were approved by local authorities (CCD, The Hague, The Netherlands) for the care and use of animals with related codes of practice. Mice were housed in IVC greenline cages and were provided with ad libitum food and water and cage enrichment.

### Clinical Observations

Gross pathology: Mice were weighed every second or third day and scored for any decrease in welfare using an in-house scoring sheet. On the day of weighing, the total body temperature was measured behind the ear by using a medisana infrared thermometer.

Organ processing and weight: On days 3, 8, and 30, mice were anesthetized using inhalation of isoflurane. Upon reaching deep anesthesia, blood was collected through retro-orbital bleeding. Blood was collected in lithium-heparin-coated tubes (Lit-Hep, Greiner, 450535) for blood biochemical analysis and in K_3_EDTA-coated tubes (Greiner, 450530) for further hematological analysis. Afterward, mice were sacrificed through cervical dislocation. The spleen, liver, kidney, lung, heart, and brain were harvested and weighed on an analytical balance. Subsequently, the spleen was cut in half, and one part was used for the analysis of the immune cell composition. The other half was fixed in 4% formalin solution (CM4094–9020, CoreMed), together with the other organs overnight, and paraffin-embedded (FFPE) for downstream histological analysis.

### Histological Analysis of Organs

Slices of 4 μm FFPE organs were cut and pasted on SuperFrost Plus slides (631–9483, Thermo Fisher Scientific). Slides were deparaffinized via immersion in xylene (4055–9005, KLINIPATH) and subsequently ethanol. After rinsing with water, slides were stained with eosin and in hematoxylin (51275–1L Sigma), rinsed with water, and immersed in xylene. Slides were enclosed with a QuickD mounting solution (Klinipath, 7281). Images were taken using a PANNORAMIC 1000 (3DHISTECH) and analyzed using the Slide Viewer 2.5 (3DHISTECH).

### Blood Hematology and Chemistry

After blood collection, lit-hep tubes were centrifuged for 10 min at 2000*g* at rt. ALAT, ASAT, blood creatinine, blood urea nitrogen (BUN), and CRP were measured directly in plasma using a Cobas C8000 (Roche). Various hematological parameters were measured in EDTA-treated full blood using an XN-10 automated hematology analyzer (Sysmex).

### Antibodies and Reagents in Flow Cytometry

For flow cytometric analysis, splenocytes were stained for viability using an eFluor 780 fixable viability dye (eBiosciences, 65–0865–014). Cells were stained using the following antibodies: αCD45.2-PerCP (BioLegend, 109826), αCD11c-BV510 (BioLegend, 117337), αCD45R-BV421 (BioLegend, 103240), αCD11b-APC (BioLegend, 101212), αLy-6G-PE/Cy7 (BioLegend, 127618), αCD3-PE (BD, 553064), αNK1.1-A488 (BioLegend, 108702), and αCD16/CD32-purified (BD, 553142). Samples were run on BD FACVerse (BD Biosciences) and analyzed using Flowlogic 8.7 after compensation using the AbC Total Antibody Compensation Bead Kit (Invitrogen, A10497).

### Antibody Titer Determination

Antibody titers were measured using the enzyme-linked immunosorbent assay. Briefly, a 96-well maxisorp plate (Thermo Fisher, 442404) was coated with 1 μg/mL bIF in 0.1 M carbonate buffer pH 9.8 solution overnight at 4 °C. After washing 3× with PBS, 0.05% CHAPS, wells were blocked with 2% NFDM in PBS for 90 min at rt and subsequently washed. Lit-Hep plasma samples were serially diluted (25×–51200×) in 0.5% NFDM PBS and added to the plate. After 90 min incubation at rt, plates were washed and incubated with HRP-conjugated Goat anti-Mouse IgG (31439, Invitrogen) for 90 min at room temperature. Plates were washed 5x with washing buffer, and 3x with PBS prior to adding a 1× TMB substrate (Invitrogen). The enzymatic reaction was stopped after 10–25 min by adding 1 M H_2_SO_4_. Plates were read on a Biorad i-Mak plate reader at a wavelength of 450 nm.

### Statistics

All data is represented as mean ± standard error of the mean (SEM). Flow cytometry data was processed using Flowlogic v8.3. Graphs were generated in GraphPad Prism (version 8.0.2). Statistical analysis was performed on transformed data where appropriate, using GraphPad Prism with the appropriate testing methods as indicated in the figure legends. Statistical significance was defined as a two-sided significance level of <0.05. Only relevant p-values <0.05 are indicated in the graphs.

## Supporting Information Available

Figure S1: additional hematological parameters in the blood of mice; Figure S2: gating strategy for spleen cell composition; Figure S3: spleen cell composition; Table S1: organ weights of mice after iv or sc injection; Table S2: number of mice scoring mild/minimal for histopathological alterations of organs (PDF)

## ABBREVIATIONS

PIC: polyisocyanopeptide;
IF: immunofilament;
PIC3eg: triethylene glycol PICs;
PIC4eg: tetra-ethylene glycol PICs;
SPAAC: strain-promoted alkyne azide click reactions;
BCN: bicyclononyne;
DBCO: dibenzocyclooctyne;
pMHC: peptide-loaded major histocompatibility complex;
bIF: blank IF;
iv: intravenous;
sc: subcutaneous;
ALAT: alanine aminotransferase;
ASAT: aspartate aminotransferase;
CRP: C-reactive protein;
H&E: hematoxylin and eosin;
MPS: mononuclear phagocytic system;
FDA: U.S. Food and Drug Administration
