# Supplementary material for: Immunofilaments Are Well Tolerated after Local or Systemic Administration in Mice
Source: ACS Pharmacol Transl Sci. 2024 May 3;7(6):1874–83. doi: 10.1021/acsptsci.4c00180 (PMC11184597; doi:10.1021/acsptsci.4c00180)
Supplement: Supplementary file 1 — pt4c00180_si_001.pdf [file pt4c00180_si_001.pdf]

Immunofilaments are well tolerated after local or systemic administration in mice

Lea Weiss‡, René Classens‡, Marjolein Schluck, Emilia Grad, Yusuf Dölen, Lieke van der Woude, Dominique van Midden, Lisa Maassen, Kiek Verrijp, Koen van Riessen, Eric van Dinther, Philipp M. Hagemann, Carl G. Figdor, Roel Hammink**

Lea Weiss, René Classens, Marjolein Schluck, Emilia Grad, Yusuf Dölen†, Lisa Maassen, Kiek Verrijp, Koen van Riessen†, Eric van Dinther, Philipp M. Hagemann, Carl G. Figdor, Roel Hammink

Department of Medical BioSciences, Radboudumc, Geert Grooteplein 26, 6525 GA Nijmegen, The Netherlands.

Lea Weiss, Carl G. Figdor

Institute for Chemical Immunology, Nijmegen 6525 GA, Netherlands

Lea Weiss, Marjolein Schluck, Yusuf Dölen, Kiek Verrijp, Carl G. Figdor, Roel Hammink

Division of Immunotherapy, Oncode Institute, Radboud University Medical Center, Nijmegen 6525 GA, Netherlands

Lieke van der Woude, Dominique van Midden

Department of Pathology, Radboudum, Geert Grooteplein 10, 6525 GA Nijmegen, The Netherlands.

Corresponding Authors

*Carl G. Figdor: Carl.Figdor@radboudumc.nl

*Roel Hammink: Roel.Hammink@radboudumc.nl

SUPPORTING INFORMATION

Figure S1. Additional hematological parameters in the blood of mice.

Figure S2. Gating strategy for spleen cell composition.

Figure S3. Spleen cell composition

Table S1. Organ weights of mice after iv or sc injection.

Table S2. Number of mice scoring mild/minimal for histopathological alterations of organs.

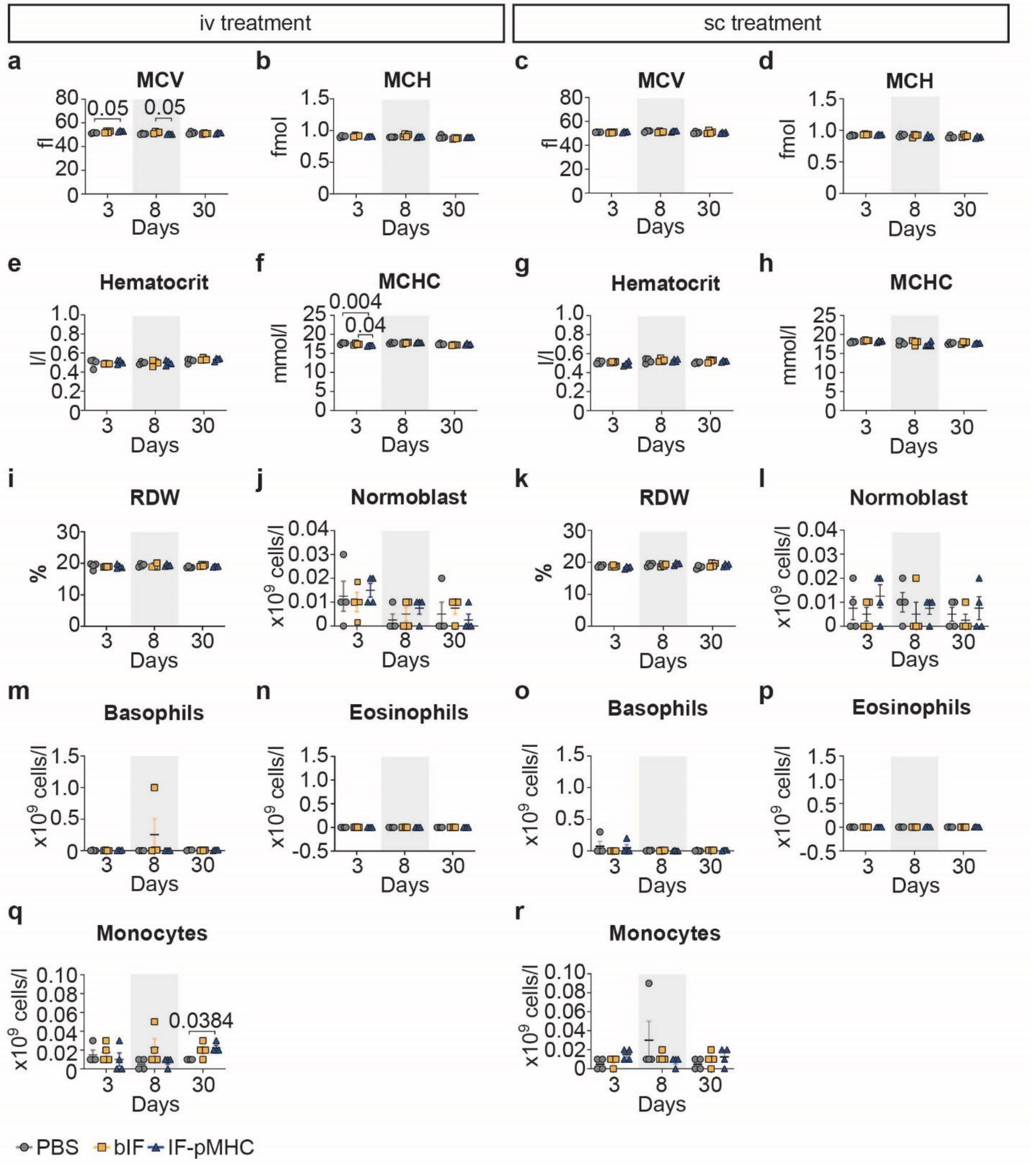

Figure S1. Additional hematological parameters in the blood of mice. Evaluation of mouse peripheral hematology 3,8 or 30 days after iv or sc injection of treatment. Statistical significance was tested per day using one-way ANOVA and post-hoc Tukey's multiple comparison test or Kruskal-Wallis test with post-hoc Dunn's test. n = 4. MCV: Mean corpuscular volume; MCH: Mean

corpuscular hemoglobin; MCHC: Mean corpuscular hemoglobin concentration; RDW: Red cell distribution width.

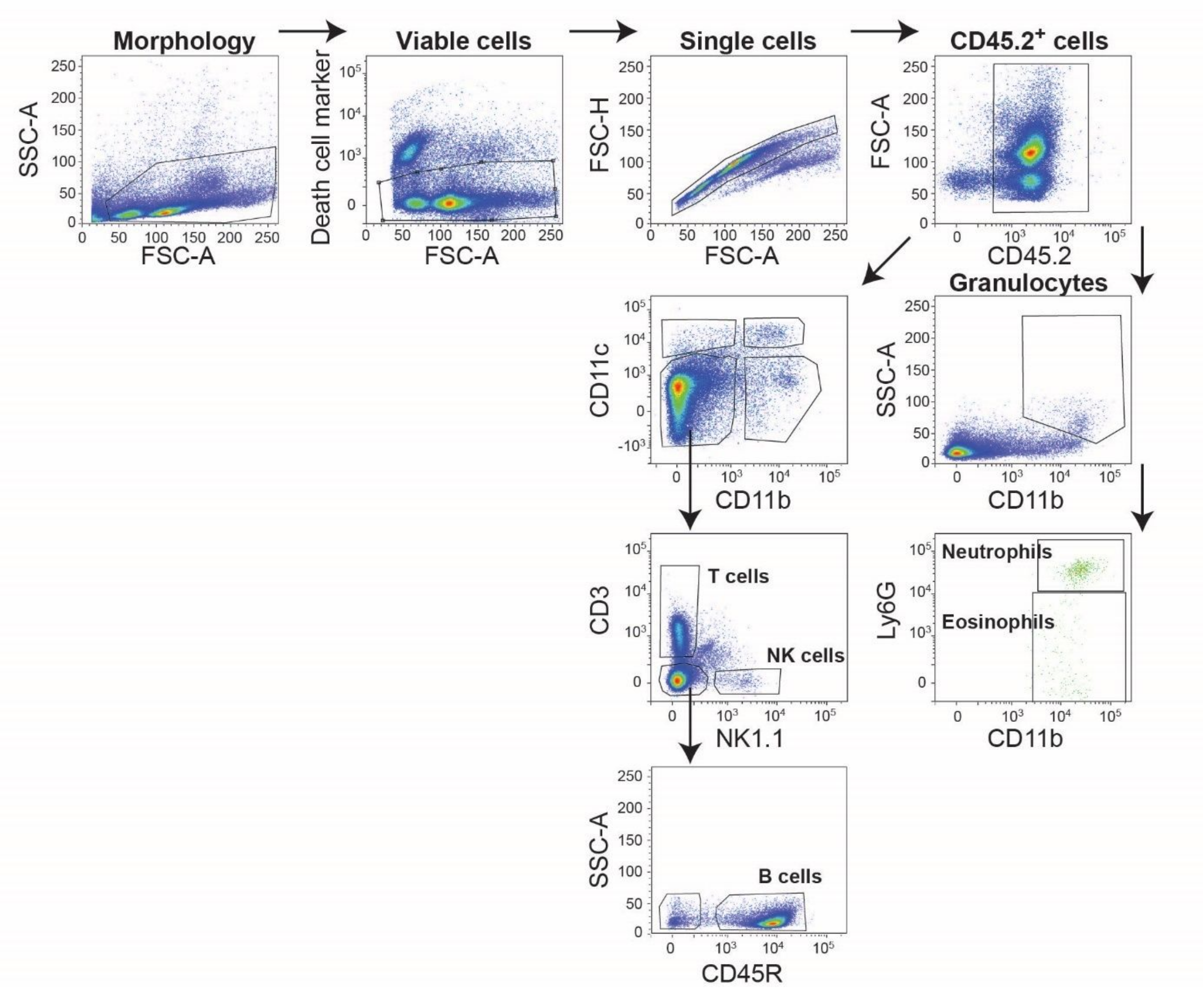

Figure S2. Gating strategy of spleen cell composition.

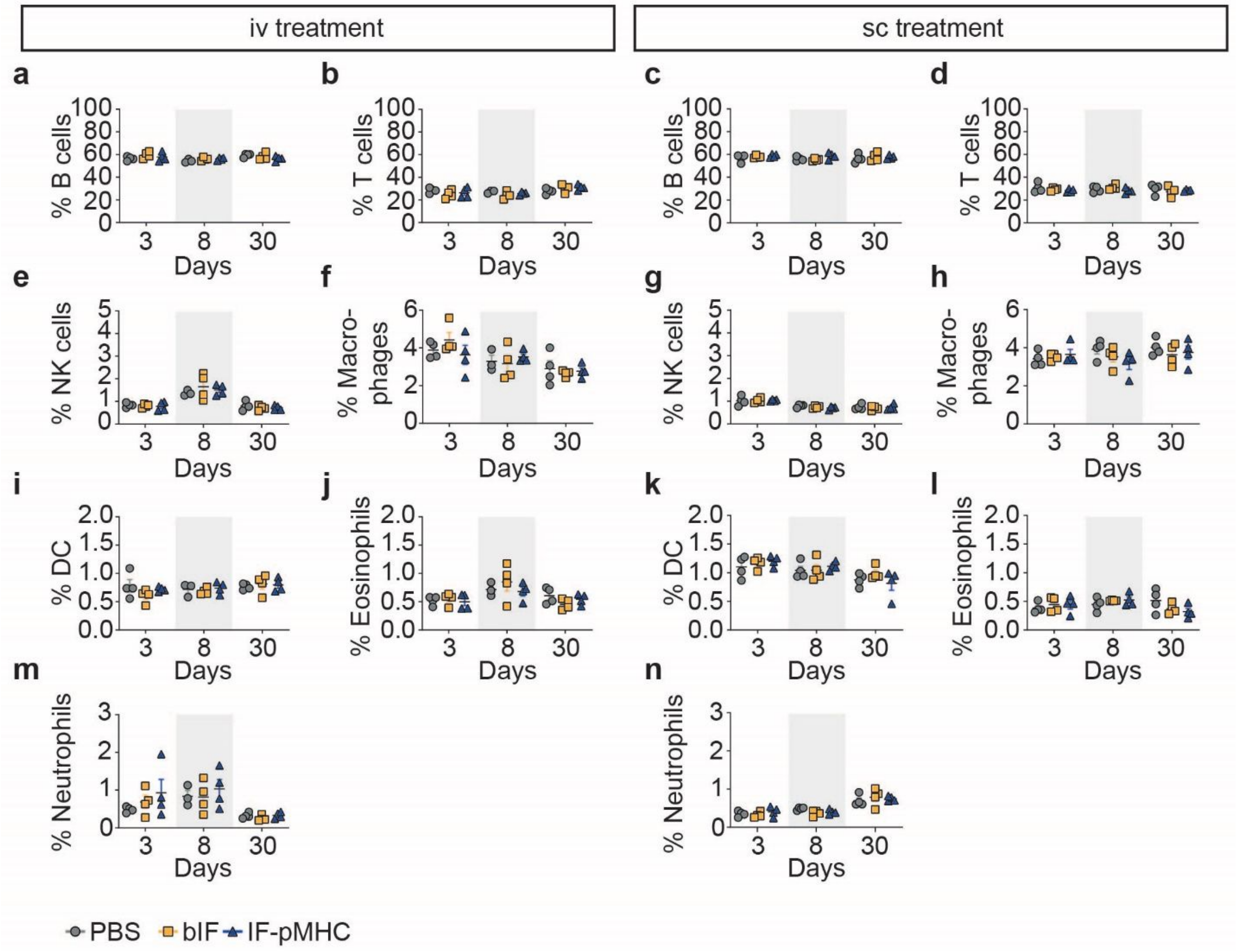

Figure S3. Spleen cell composition. (a-n) Flow cytometric evaluation of the percentage of different cell populations in the spleen on day 3, day 8 or day 30 following iv treatment or sc treatment with PBS, bIF or IF-pMHC. Statistical significance was tested per day using one-way ANOVA and Tukey's multiple comparison test, or Kruskal-Wallis test with post-hoc Dunn's test. DC: dendritic cell; NK cell: Natural Killer cell

Table S1: Organ weights of mice after iv or sc injection. Mice were treated iv with PBS, bIF or IF-pMHC and organs were taken out on day 3, 8 and 30 and weighted. Data is shown as mean ± SEM. n = 3-4.

	Intravenous	PBS	bIF	IF-pMHC
Day 3	Spleen	103.6 ± 3	104 ± 4.1	103.6 ± 7.7
	Liver	1354.5 ± 24.1	1367 ± 32.7	1385.9 ± 87.9
	Kidney	161.6 ± 3.6	159.4 ± 14.3	170.3 ± 12.2
	Heart	140.6 ± 10.1	144.6 ± 10.8	144.7 ± 3.5
	Lung	230.1 ± 10.7	198.6 ± 14.9	237.5 ± 20.3
	Brain	477.0 ± 21.2	519.3 ± 14.1	461.8 ± 45.2
Day 8	Spleen	102.2 ± 6.2	118.3 ± 8.0	93.9 ± 4.8
	Liver	1252.1 ± 67.3	1252.3 ± 128.1	1185.4 ± 31.6
	Kidney	156.9 ± 10	185 ± 8.1	144.7 ± 7.4
	Heart	127.4 ± 5.8	144.5 ± 10.9	126.1 ± 5.9
	Lung	191.8 ± 10.8	237.5 ± 18.6	217.2 ± 21.9
	Brain	509.3 ± 21.5	522.1 ± 22.8	494.4 ± 2.3
Day 30	Spleen	97.7 ± 6.9	105.9 ± 10.6	101.1 ± 2.5
	Liver	1384.2 ± 122.8	1416.6 ± 47	1493.9 ± 33.6
	Kidney	177.1 ± 11.2	161 ± 6.3	180 ± 4.4
	Heart	143.9 ± 4.2	135.0 ± 5.5	137.9 ± 8.1
	Lung	231.4 ± 9.2	252.3 ± 18.3	278.8 ± 26.4
	Brain	547.2 ± 4.8	535.6 ± 19.7	552 ± 13.6

	Subcutaneous	PBS	bIF	IF-pMHC
Day 3	Spleen	111.6 ± 6.3	97.6 ± 4.2	95.9 ± 6.2
	Liver	1334.5 ± 51.6	1315.8 ± 36	1350.6 ± 75.6
	Kidney	149.5± 8.5	155.5 ± 8.4	161.9 ± 9.3
	Heart	127.1 ± 7.2	130.0 ± 5.3	139.1 ± 8.1
	Lung	200.4 ± 11.8	209.8 ± 13.8	226.2 ± 22.1
	Brain	512.6 ± 7.8	533.4 ± 16.2	505.6 ± 13.8
Day 8	Spleen	100.0 ± 3.1	90.8 ± 8.5	100.4 ± 6.9
	Liver	1387 ± 42.8	1408.9 ± 18.2	1443.1 ± 8.5
	Kidney	164.4 ± 11.6	170.3 ± 17.9	169.4 ± 3.3
	Heart	130.2 ± 8	123.5 ± 4	150.6 ± 11.4
	Lung	233.1 ± 19.9	209.1 ± 6.8	233.6 ± 3.7
	Brain	520.4 ± 14.6	506.0 ± 13.7	522.0 ± 19.7
Day 30	Spleen	113.6 ± 4.9	124.2 ± 5.1	116.3 ± 6.6
	Liver	1494 ± 46.2	1430.9 ± 83	1464.3 ± 37.6
	Kidney	183.1 ± 2.9	177.8 ± 14.5	175.4 ± 5.9
	Heart	141.9 ± 8.7	149 ± 11	155.9 ± 9.8
	Lung	251.6 ± 11.1	229.1 ± 12.8	219.5 ± 9.1
	Brain	576.2 ± 16	528.1 ± 19.7	543.9 ± 18.9

Table S2: Number of mice scoring mild/minimal for histopathological alterations of organs. Mice were treated iv with PBS, bIF or IF-pMHC and organs were taken out on day 3, 8 and 30 and prepared for histopathological analysis. Histology slides were evaluated and scored under blinded conditions by two independent pathologists. None of the mice had a higher scoring than minimal/mild (=1).

	Scoring degree (1-3):	1								
		Day 3			Day 8			Day 30		
		PBS	bIF	IF-pMHC	PBS	bIF	IF-pMHC	PBS	bIF	IF-pMHC
**Spleen**		0	0	0	0	0	0	0	0	0
**Liver**	Increased cellularity	0	1	0	1	2	0	0	1	0
	Mononuclear cell infiltration	1	1	0	1	2	0	0	1	0
	Polynuclear cell infiltration	1	1	0	0	1	0	0	0	0
	Vacuolisation/ steatosis	2	0	0	0	0	0	0	0	0
	Apoptosis	0	1	0	0	2	1	0	0	1
	Trombi	1	0	0	0	0	0	0	0	0
	Necrosis	0	0	0	0	0	0	0	0	0
**Lung**	Increased cellularity	2	0	0	2	2	0	0	0	1
	Mononuclear cell infiltration	2	0	0	2	2	0	0	0	1
	Polynuclear cell infiltration	0	0	0	0	1	0	0	0	0
	Trombi	0	0	0	0	0	0	0	0	0
	Necrosis	0	0	0	0	0	0	0	0	0
**Kidney**	Increased cellularity	1	1	0	3	3	1	1	1	1
	Mononuclear cell infiltration	1	1	0	3	3	0	1	1	1
	Polynuclear cell infiltration	0	0	0	0	0	0	0	0	0
	Tubulopathic changes	0	0	0	0	0	0	0	0	0
	Trombi	0	0	0	0	0	0	0	0	0
	Necrosis	0	0	0	0	0	0	0	0	0
